# β-Glucan Extracted from Highland Barley Alleviates Dextran Sulfate Sodium-Induced Ulcerative Colitis in C57BL/6J Mice

**DOI:** 10.3390/molecules26195812

**Published:** 2021-09-25

**Authors:** Minjie Chen, Shuhua Tian, Shichao Li, Xinyi Pang, Jing Sun, Xiaoyu Zhu, Fengxia Lv, Zhaoxin Lu, Xiangfei Li

**Affiliations:** 1 College of Food Science and Technology, Nanjing Agricultural University, Nanjing 210095, China; 2020808101@stu.njau.edu.cn (M.C.); 2019808139@stu.njau.edu.cn (S.L.); zhuxiaoyu@njau.edu.cn (X.Z.); 2 Collaborative Innovation Center for Modern Grain Circulation and Safety, Key Laboratory of Grains and Oils Quality Control and Processing, College of Food Science and Engineering, Nanjing University of Finance and Economics, Nanjing 210023, China; tianshuhua88@outlook.com (S.T.); pangxinyi@nufe.edu.cn (X.P.); jingsun@nufe.edu.cn (J.S.)

**Keywords:** highland barley β-glucan, ulcerative colitis, inflammatory cytokines, intestinal tract barrier, intestinal flora

## Abstract

Inflammatory bowel disease (IBD), which significantly affects human health, has two primary presentations: Crohn’s disease and ulcerative colitis (UC). Highland barley is the most common food crop for Tibetans and contains much more β-glucan than any other crop. Highland barley β-glucan (HBBG) can relieve the gastrointestinal dysfunction and promote intestines health. This study aimed to evaluate whether HBBG can relieve UC in mice. A mouse model of UC was established by adding 2% dextran sulfate sodium (DSS) to drinking water for 1 week. UC was alleviated after the introduction of the HBBG diet, as indicated by reductions in the disease activity index (DAI) score, histopathological damage, and the concentration of colonic myeloperoxidase (MPO), along with an improvement in colonic atrophy. Furthermore, we found that HBBG can increase the relative transcriptional levels of genes encoding ZO-1, claudin-1, occludin, and mucin2 (MUC2), thereby reducing intestinal permeability. Additionally, HBBG maintained the balance of proinflammatory and anti-inflammatory cytokines and modulated the structure of the intestinal flora.

## 1. Introduction

Ulcerative colitis (UC) is a common inflammatory bowel disease (IBD) characterized by inflammation of the mucosa and submucosa from the rectum to the appendix [1]. With the industrialization and urbanization of society, individuals’ diets, occupations, lifestyles, and exposures to drugs, pollution, and microbes are also constantly changing. As a result, the UC morbidity rate has been increasing worldwide, indicating that UC has become a global issue [2]. Although the pathogenic mechanism of UC remains unclear, it is conceived to involve genetic and environmental factors [3]. Among various environmental factors, diet plays an important role in the pathogenesis of UC [4]. Currently, aminosalicylic acid, corticosteroids, and immunosuppressive agents are prescribed to treat UC [5]. However, headaches, nasopharyngitis, systemic infections, and many other adverse reactions may occur in response to these treatments [6,7]. Therefore, it is important to find effective treatments with fewer side-effects.

In recent years, the extraction of active substances from biological resources has attracted widespread interest. It has been established that pectin, chitosan, fructan, algal polysaccharides and other natural polysaccharides can exert therapeutic effects on UC. These polysaccharides are thought to balance inflammatory factors, regulate intestinal flora, protect the intestinal mucosa, and regulate the immune system. Moreover, they have the advantage of being safe and effective, with few side-effects [5]. As a polysaccharide, β-glucan is formed by combining d-glucose molecules via β-glycosidic bonds, and it mainly exists in fungal and plant cell walls. β-Glucan found in grains such as rye, barley, and oats mainly consists of β-1,3 and β-1,4 glycosidic polysaccharides [8]. Meanwhile, β-1,3-glucan and β-1,6-glucan constitute the β-glucan found in fungi [9]. Brown et al. demonstrated that β-glucan polymerized via β-1,3-glycosidic bonds can strengthen the mammalian innate immune system [10]. Previous research has shown that yeast β-glucan can improve UC and reduce intestinal permeability by inhibiting the expression of related inflammatory mediators and increasing the relative transcriptional levels of genes encoding tight junction proteins [11]. Shi et al. found that *Lentinus* β-glucan exerted anti-inflammatory effects through modulation of related pathways [12]. Liu et al. showed that oral oat β-glucan inhibited the mRNA and protein levels of proinflammatory cytokines and inducible nitric oxide synthase in dextran sulfate sodium (DSS)-induced UC in mice, suggesting that it may exert anti-inflammatory effects [13].

As the only crop that can grow more than 4200 m above sea level, highland barley has a high nutritional value and contains a large amount of biologically active substances [14,15]. A comparison between highland barley and other cereals is shown in Table S1 [16]. More than 100 barley varietals from China, Canada, and Australia were determined by Zhang et al., and they found that Chinese highland barley contained the highest level of β-glucan. It is thought that the growing environment significantly influences the β-glucan content of highland barley [17]. Highland barley grown at high altitudes contains more β-glucan and has improved biological activity compared with that grown at low altitudes [18]. Recent studies about highland barley have mainly focused on its antioxidant activity, as well as its abilities to regulate intestinal flora and blood lipid levels and prevent metabolic syndrome [14,19,20]. However, the effect of highland barley β-glucan (HBBG) on UC has not been extensively studied. Recent studies have demonstrated that HBBG can alleviate gastric damage caused by ethanol and promote intestinal health. HBBG has been reported to improve colon atrophy and increase the intestinal concentration of total short-chain fatty acids (SCFAs). Moreover, HBBG can decrease intestinal pH and the abundance of proinflammatory intestinal bacteria [20,21]. These studies indicate that HBBG may have a potential therapeutic effect on UC. Therefore, in this study, DSS was used to induce UC in mice [22], and HBBG was added to the diet fed to the mice. Changes in intestinal tissues, inflammatory cytokine expression, intestinal barrier function, and intestinal flora were monitored in mice, allowing us to examine the therapeutic effects of HBBG on UC.

## 2. Results

### 2.1. The Effects of HBBG on Histopathological Indicators

**Figure 1:**
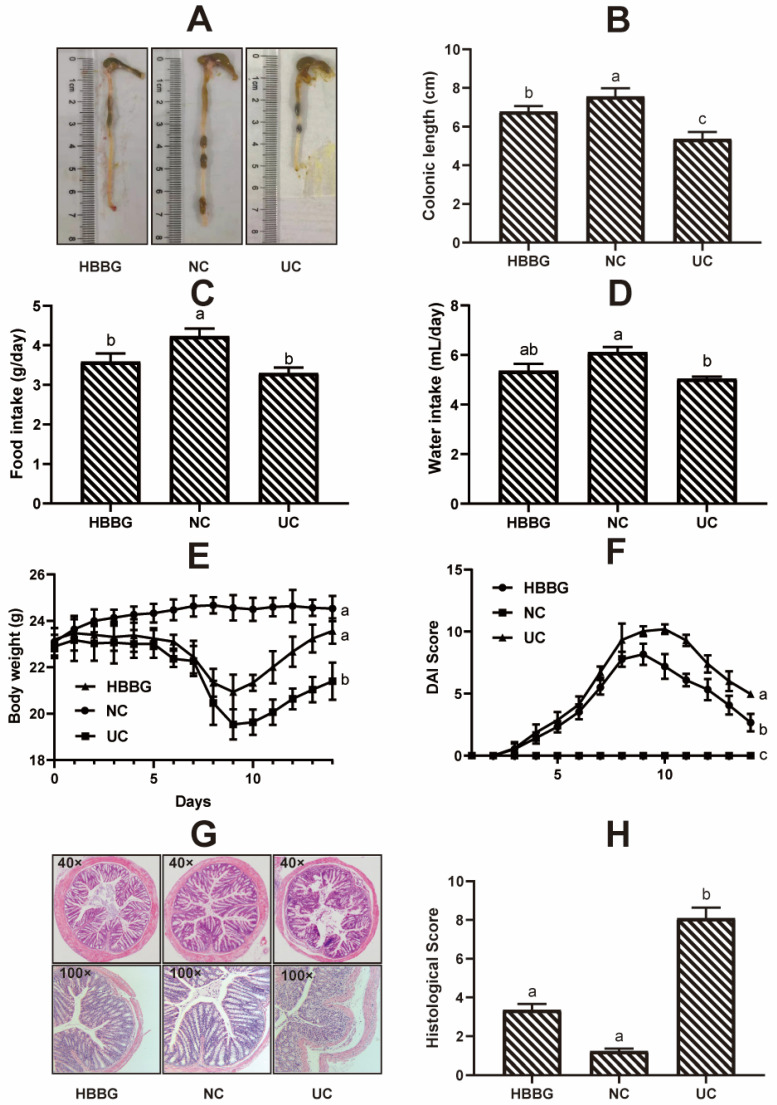
The effects of HBBG on histopathological indicators: (**A**) macroscopic pictures of colons; (**B**) colonic length; (**C**) food intake; (**D**) water intake; (**E**) body weight; (**F**) disease activity index (DAI); (**G**) histological examination; (**H**) colonic histological injury score. Different letters indicate statistically significant differences (*p* < 0.05); on the contrary, the same letters indicate no statistically significant differences (*p* > 0.05).

Representative images of colons in the three groups are shown in Figure 1A. The differences in colon length between the experimental groups are shown in Figure 1B. Compared with the normal control (NC) group, mice in the UC group had significantly shorter colons (*p* < 0.05), indicating successful UC induction. After the HBBG diet, colonic atrophy was improved to a certain extent. Although the colons of mice in the HBBG group were significantly shorter (*p* < 0.05) than those of mice in the NC group, they were significantly longer (*p* < 0.05) than those of mice in the UC group. The results above indicates that dietary HBBG can prevent the colon shortening in UC mice.

The food intake, water intake, and body weight are shown in Figure 1C–E. The DAI scores of the mice are shown in Figure 1F. No mice died during the experiment. The DAI score of the NC group was maintained at zero over the course of the experiment, indicating that the mice remained in good health. In contrast, during the induction of UC, the DAI score of mice in the UC and HBBG groups increased gradually and reached a peak on day 9 or day 10. The DAI score of the UC mice gradually decreased from day 10 to day 14, suggesting that the mice possess an intrinsic ability to recover from UC to a certain extent upon removal of DSS exposure. The DAI score decreased at a faster rate in the HBBG mice compared to the UC mice. The DAI score was consistent with colon length. Together, these findings indicate that HBBG can improve UC in mice.

Histopathological sections of colon stained with hematoxylin and eosin are shown in Figure 1G, and the corresponding histological scores are shown in Figure 1H. The mice in the NC group had complete colonic epithelial structures, neatly arranged colon villi, abundant goblet cells and absorptive cells, and intact crypts, with no inflammatory cell infiltration. The histological score was 1.23 ± 0.13. In the UC group, significant damage to the epithelial structure and severe edema were observed. The crypts, villi, and other structures were deformed, and inflammatory cell infiltration was noted. The histological score was 8.09 ± 0.55, which was significantly higher (*p* < 0.05) than that of the NC group. In the HBBG group, the epithelial structure was restored. Crypts and colon villi were arranged neatly, and the majority of the goblet cells were retained, with no signification of inflammatory cell infiltration observed. The histological score was 3.35 ± 0.31, which was significantly lower (*p* < 0.05) than that of the UC group. In addition, the differences in histological scores between the NC group and the HBBG group were not significant. These results further suggest that HBBG can improve UC-induced pathological damage in mouse colon tissue.

### 2.2. The Effects of HBBG on the Intestinal Barrier

**Figure 2:**
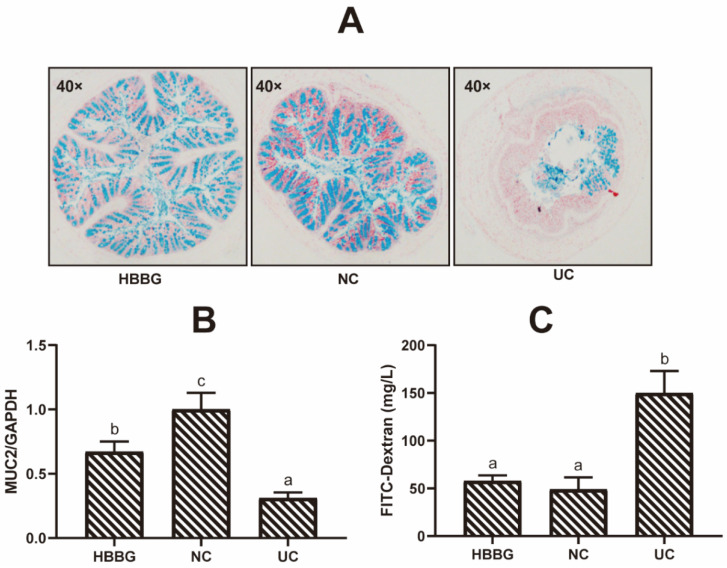
The effects of HBBG on intestinal permeability: (**A**) Alcian blue staining; (**B**) the relative transcriptional levels of the gene encoding MUC2; (**C**) the concentration of Dextran-FITC in the blood samples. Different letters indicate statistically significant differences (*p* < 0.05); on the contrary, the same letters indicate no statistically significant differences (*p* > 0.05).

As can be seen in the Alcian blue staining images in Figure 2A, there were abundant goblet cells present in the colon of the NC group. The epithelial cells were tightly structured and neatly arranged. Moreover, the epithelial surface was protected by a mucus layer. The goblet cells of mice in the UC group were significantly damaged, and the number of goblet cells present in the intestinal crypts was extremely low. The mucus layer covered only part of the intestinal epithelial tissue. In the HBBG group, a relatively complete mucus layer was seen, and goblet cells were abundant. These results suggest that HBBG can promote mucus layer formation, thereby enhancing intestinal resistance to microorganisms.

The relative transcriptional levels of the gene encoding MUC2 in the intestines of mice in the three groups are shown in Figure 2B. Compared with the NC group, transcription of MUC2 was significantly downregulated in the UC group (*p* < 0.05). Although transcriptional levels of MUC2 in the HBBG group were lower than those in the NC group, they were significantly higher (*p* < 0.05) than those in the UC group.

Intestinal permeability was determined by measuring the amount of FITC-Dextran transferred from the intestinal tract to the blood (Figure 2C). The FITC-Dextran content in the blood of the UC group mice was significantly higher (*p* < 0.05) than that in the other two groups, reaching up to 149.87 ± 23.16 mg/L. Although the FITC-Dextran content in the HBBG group was slightly higher than that in the NC group, this difference was not significant.

**Figure 3:**
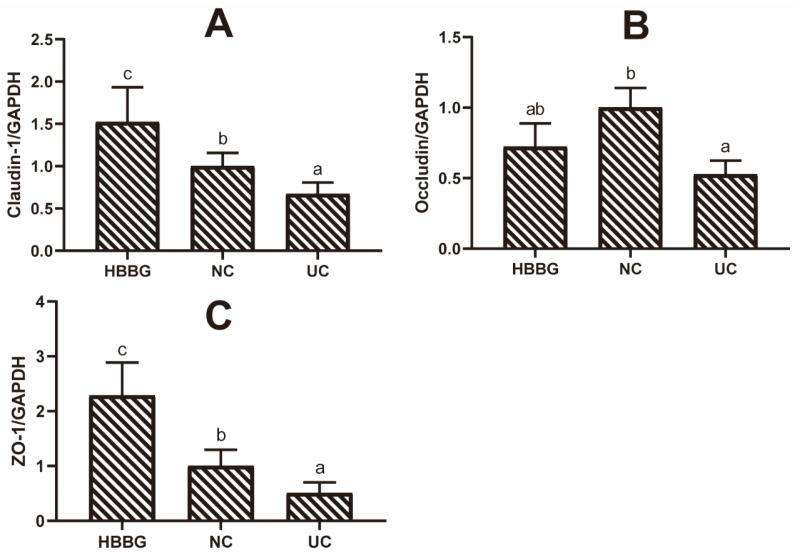
The effect of HBBG on mRNA expression levels of tight junction-related proteins: (**A**) claudin-1; (**B**) occludin; (**C**) ZO-1. Different letters indicate statistically significant differences (*p* < 0.05); on the contrary, the same letters indicate no statistically significant differences (*p* > 0.05).

To analyze the effect of HBBG on the abundance of tight junction proteins, the relative transcriptional levels of genes encoding claudin-1, occludin, and ZO-1 were measured in colon tissue (Figure 3A–C). The transcription of all three genes was significantly downregulated in the UC group (*p* < 0.05). However, the HBBG diet restored the transcriptional levels of these genes (*p* < 0.05). These results indicate that HBBG can reduce intestinal permeability to restore the barrier function of the intestinal tract.

### 2.3. The Effects of HBBG on Concentration of MPO

**Figure 4:**
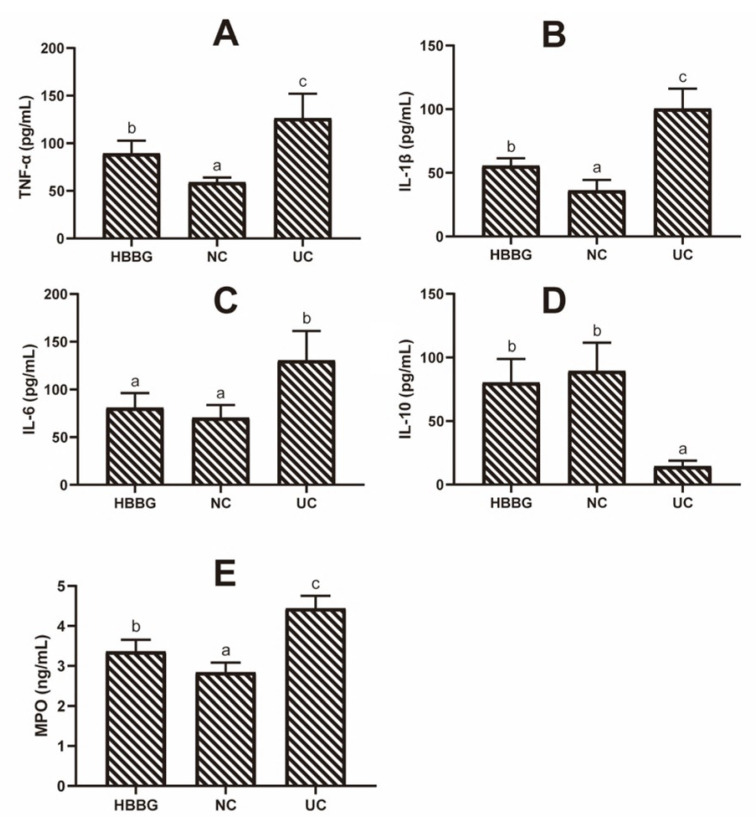
The effects of HBBG on serum inflammatory cytokines and MPO: (**A**) TNF-α; (**B**) IL-1β, (**C**) IL-6; (**D**) IL-10; (**E**) MPO. Different letters indicate statistically significant differences (*p* < 0.05); on the contrary, the same letters indicate no statistically significant differences (*p* > 0.05).

MPO, mainly secreted by neutrophils and monocytes, is a biomarker of inflammation. The concentration of MPO in the colon tissues of different groups is presented in Figure 4E. The MPO level of mice in the NC group was only 2.84 ± 0.24 ng/mL, compared with 4.44 ± 0.31 ng/mL in the UC mice. The concentration of MPO in the HBBG group was 4.44 ± 0.31 ng/mL, which was significantly lower than that in the UC group (*p* < 0.05). The results above indicate that HBBG can reduce inflammation in UC mice.

### 2.4. The Effects of HBBG on Serum Inflammatory Cytokines

The changes in proinflammatory and anti-inflammatory cytokines play a crucial role in the pathogenesis of UC. ELISA kits were used to quantify the serum concentrations of inflammatory cytokines among the different groups. As shown in Figure 4, DSS treatment significantly increased (*p* < 0.05) the concentrations of proinflammatory cytokines TNF-α, IL-1β, and IL-6 to 126.28 ± 24.81, 100.45 ± 15.61, and 130.51 ± 30.67 pg/mL, respectively. Moreover, DSS significantly reduced (*p* < 0.05) the concentration of the anti-inflammatory cytokine IL-10 in UC mice (14.45 ± 4.42 pg/mL) by 79.39% compared with that in the NC group (89.56 ± 22.1 pg/mL). In the HBBG group, the concentrations of TNF-α, IL-1β, and IL-6 were 89.28 ± 13.43, 55.56 ± 5.89, and 80.99 ± 15.33 pg/mL, respectively, representing reductions of 29.30%, 44.69%, and 37.94%, compared with the values in UC mice. Meanwhile, the concentration of IL-10 (80.33 ± 18.47 pg/mL) increased by approximately 456% in the HBBG mice relative to the UC mice. These findings suggest that HBBG can balance the concentrations of anti-inflammatory and proinflammatory cytokines in serum.

### 2.5. The Effects of HBBG on Intestinal Flora

**Figure 5:**
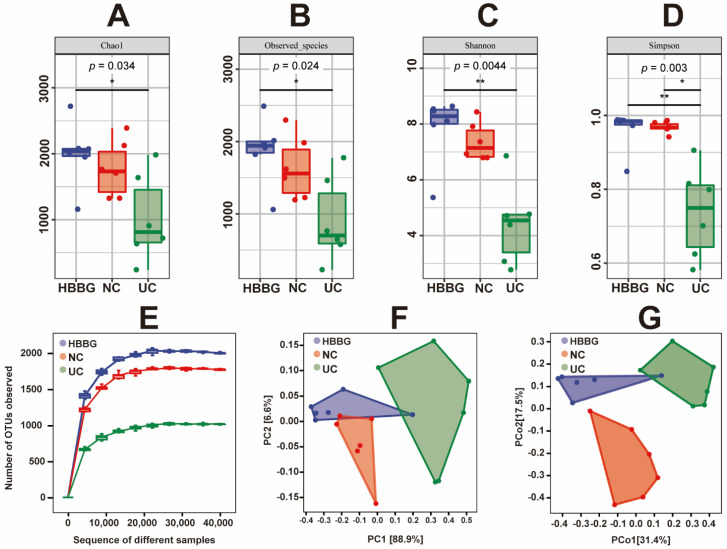
The effects of HBBG on the alpha and beta diversity of intestinal flora: (**A**) Chao1 index; (**B**) observed species index; (**C**) Shannon index; (**D**) Simpson index; (**E**) rarefaction curves; (**F**) PCA; (**G**) PCoA. * *p* < 0.05, ** *p* < 0.01.

Rarefaction curves allow a comparison of the richness of species in different samples and indicate whether the sequencing depth is sufficient. As shown in Figure 5E, the number of operational taxonomic units (OTUs) increased significantly at first and then leveled off, with more samples only generating a few new OTUs. This indicated that the sequencing depth was reasonable. The UC samples were located at the bottom, suggesting that the samples have a less rich intestinal flora than those from the other groups of mice. The HBBG samples exhibited improved intestinal microbe richness, with values even higher than those from the NC group.

The alpha diversity of all samples is shown in Figure 5A–D. The Chao1 index and observed species index can represent the richness of the flora in the samples. In other words, DSS appeared to reduce the richness of intestinal flora. In contrast, the HBBG intervention significantly increased (*p* < 0.05) the richness of intestinal flora. Additionally, the richness of intestinal flora was higher in the HBBG group than in the NC group. The Shannon index and Simpson index represent the diversity and uniformity of the flora in the samples. DSS reduced both indices, indicating that it can decrease both the diversity and the uniformity of the intestinal flora. Conversely, HBBG treatment increased both indices.

To analyze the beta diversity of the intestinal flora, PCA and PCoA were performed on all samples. The PCA and PCoA results are shown in Figure 5F–G. The NC group and the UC group could be completely separated, suggesting that DSS can alter the intestinal microbiome structure.

**Figure 6:**
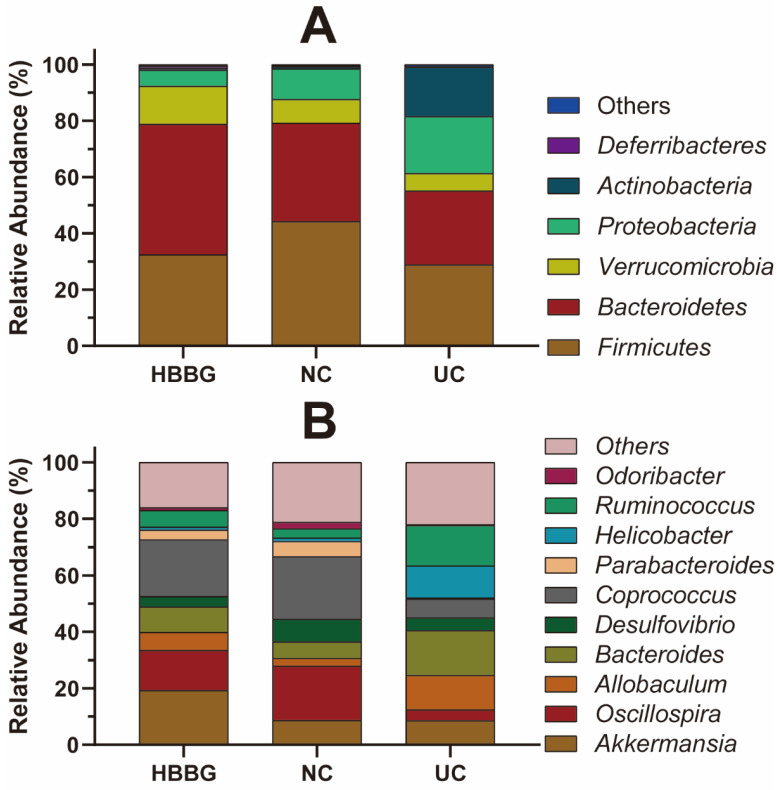
The effects of HBBG on intestinal flora: (**A**) phylum level; (**B**) genus level.

Additionally, we analyzed the relative abundances of intestinal flora at the phylum and genus levels. Figure 6A displays the relative abundances of the intestinal flora at the phylum level in different groups. The relative abundances of Firmicutes, Bacteroidetes, and Verrucomicrobia in the UC group decreased to different degrees compared with the NC group and the HBBG group, while the relative abundances of Proteobacteria and Actinobacteria increased. The relative abundance of Bacteroidetes and Verrucomicrobia increased in the HBBG group compared with the NC group, whereas the relative abundance of Firmicutes and Proteobacteria decreased.

Figure 6B shows the relative abundances of intestinal flora at the genus level in different groups. Ten bacterial genera were found to dominate. The relative abundances of *Allobaculum*, *Bacteroides*, *Helicobacter*, and *Ruminococcus* in the UC group increased to varying degrees compared with the NC group, whereas those of *Oscillospira*, *Desulfovibrio*, *Parabacteroides*, *Odoribacter*, and *Coprococcus* decreased. The relative abundances of *Oscillospira*, *Allobaculum*, *Bacteroides*, *Coprococcus*, *Parabacteroides*, *Ruminococcus*, and *Odoribacter* in the HBBG group fell to levels between those of the UC and NC groups, indicating that HBBG can restore the relative abundance of intestinal flora to normal levels. Interestingly, the relative abundance of *Akkermansia muciniphila* in the HBBG group was higher than that in the UC and NC groups.

## 3. Discussion

Establishing a successful mouse model of UC is the first step toward understanding the symptoms of UC and the effects of novel treatments. The changes in histopathology, intestinal microflora, and mucosal immunity in the mouse model induced by DSS are consistent with the clinical symptoms of human UC. Therefore, it can be used as a reliable model for studying UC [22]. In this study, UC was induced by adding 2% DSS to the daily drinking water of C57BL/6J mice for a week. The DAI score is one of the indicators that can more intuitively reflect the severity of UC. Hematoxylin and eosin staining allows the examination of histopathological changes in tissues and is one of the most effective methods used to observe local tissue damage. MPO, an oxidative stress-related enzyme found in neutrophils and other cells, can be used to quantitatively evaluate the severity of acute colitis and the efficacy of drugs in animal models of inflammation [23]. In this study, after the introduction of an HBBG diet, the DAI score of the mice gradually decreased, and the MPO content in that group was significantly lower (*p* < 0.05) than that in the UC group. HBBG also prevented colonic shortening and significantly reduced (*p* < 0.05) the tissue damage score. All these results suggest that HBBG can relieve UC.

The two mucus layers covering the surface of mouse intestinal epithelial cells are composed of MUC2. The inner mucus layer separates bacteria from the intestinal epithelial cells [24]. It was previously reported that mice with mutations in the gene encoding MUC2 develop spontaneous enteritis and diarrhea [25]. In this study, changes in the mucus layer were examined using Alcian blue staining. It was found that the mucus layer of the UC group mice was significantly diminished, such that it protected only some of the intestinal epithelial cells. After the introduction of the HBBG diet, a relatively complete mucus layer was observed on the surfaces of intestinal epithelial cells. Quantification of FITC-Dextran uptake revealed that HBBG reduced intestinal permeability. Additionally, the relative transcriptional levels of the gene encoding MUC2 in the HBBG group were significantly higher (*p* < 0.05) than those in the UC group. Claudin-1, occludin, and ZO-1 are essential components of tight junctions, which completely block the paracellular pathways between intestinal epithelial cells, thereby reducing intestinal permeability [26]. As a result, microorganisms are blocked out of the intestinal epithelial cells. Accordingly, the reactions between microorganisms and immune cells are also reduced. This study shows that adding HBBG to the diet can upregulate the transcriptional levels of genes encoding MUC2, claudin-1, occludin, and ZO-1. Therefore, it can be inferred that HBBG can alleviate UC by enhancing the barrier function of colonic epithelium.

Cytokines also play a crucial role in the pathogenesis of UC. An imbalance of pro- and anti-inflammatory cytokines can lead to impaired immune function. Due to the pleiotropy of cytokines and several signaling pathways involved, their influence on the pathogenesis of UC is quite complicated [12,27]. For example, macrophage-, dendritic cell-, and T cell-derived TNF-α plays a central role in the pathogenesis of UC and can stimulate the production of proinflammatory cytokines and induce T cells resistant to apoptosis [28]. TNF-α and IL-1β can increase intestinal tract permeability by downregulating ZO-1 and occludin expression, respectively, promoting inflammation [29,30]. IL-6 is a cytokine with dual functions (anti-inflammatory and proinflammatory) [31]. Researchers found that the plant *Ilex kudingcha* can alleviate IBD by reducing the expression and secretion of IL-1β, IL-6, and TNF-α [32]. As we all know, IL-10 is an anti-inflammatory cytokine. IL-10 can reduce the intestinal tract permeability by neutralizing IFN-γ, thereby alleviating inflammation [33]. In this study, we measured the concentrations of inflammatory cytokines in serum. After the introduction of HBBG to the diet, the concentrations of IL-1β, IL-6, and TNF-α decreased, whereas the concentration of IL-10 increased. Thus, HBBG may exert an anti-inflammatory effect by regulating inflammatory cytokines and related pathways.

Intestinal microbes are directly in contact with the intestinal barrier and are closely related to the health of the host. Researchers found that many diseases are caused by a structural imbalance of the intestinal flora [34,35]. UC is thought to occur due to imbalances in the intestinal microbiome, rather than perturbations involving a single pathogenic microorganism. On the one hand, UC leads to reduced abundances of Bacteroidetes, Lachnospiraceae, *Clostridium leptum*, *Clostridium coccoides*, *Faecalibacterium prasnitzii*, and *Bifidobacteria*. On the other hand, it can increase the abundances of Actinobacteria and Proteobacteria [36,37]. In this study, DSS led to decreases in both the richness and the diversity of intestinal flora. After treatment with HBBG, the mice exhibited higher levels of intestinal flora richness and diversity than those in the NC group. The Firmicutes/Bacteroidetes ratio in HBBG group was the lowest among the three groups. Therefore, we speculate that HBBG can reduce the Firmicutes/Bacteroidetes ratio and inhibit inflammation, which is consistent with the results of other studies [38]. *A. muciniphila* is a Gram-negative anaerobic bacterium belonging to Verrucomicrobia. It exists in the intestines of mice and humans and can use the intestinal epithelial mucosa as its sole source of carbon, nitrogen, and energy. After mucin is decomposed by *A. muciniphila*, several beneficial small-molecule metabolites are produced [39,40]. Several studies have shown that *A. muciniphila* is negatively correlated to obesity, diabetes, and other metabolic disorders [41,42]. Last year, researchers found that the pasteurized *A. muciniphila* or one of its specific outer membrane proteins can alleviate colitis and prevent the occurrence of colitis-associated colorectal cancer by modulating the levels of CD8^+^ cytotoxic T lymphocytes [43]. *A. muciniphila* can also exert an anti-inflammatory effect by preventing neutrophil infiltration and reducing intestinal inflammation [44]. In this study, HBBG intake significantly increased the relative abundance of *A. muciniphila* in the intestine. It is possible that *A. muciniphila* can speed up the metabolism of mucus layer, thereby enhancing the barrier function of the intestine. *Odoribacter* is also beneficial for IBD, and it was considered to alleviate Crohn’s disease and UC [45]. In this study, HBBG restored the relative abundance of *Odoribacter* to a certain extent. Some researchers proposed that *Allobaculum* can help the formation of the intestinal mucus layer and enhance the intestinal barrier function [46]. However, other researchers found that the mucus permeability of the mouse intestine increases as the relative abundance of *Allobaculum* increases [47]. In this study, DSS could increase the relative abundance of *Allobaculum*, while HBBG could reduce the relative abundance of *Allobaculum*. It can be inferred that the abundance of *Allobaculum* is negatively correlated with intestinal permeability. All microorganisms of the genus *Coprococcus* can promote the production of short-chain fatty acids, which is good for the health of the host [48]. Kelly et al. also found that a low abundance of *Coprococcus* can promote the development of IBD, which is also consistent with the results of this experiment [49]. Lam et al. found that the abundance of *Oscillospira* affects the barrier function of the colon. In this study, we found that the abundance of *Oscillospira* is positively correlated with the intestinal barrier function, and HBBG can alleviate the decrease in abundance of *Oscillospira* caused by DSS [50]. *Ruminococcus* can affect metabolism and promote the development of inflammation [51]. In this study, HBBG showed a good effect on inhibiting the increase of *Ruminococcus*. *Helicobacter* can mediate inflammation through NOD1 and ALPK1 on its cell wall [52]. We found that HBBG can also reduce its abundance in the mouse intestine. In summary, HBBG appears to alleviate UC by regulating the composition of the intestinal microbiome.

Due to the lack of a digestive enzyme of β-glucan in the human body, β-glucan cannot be quickly degraded. During the digestion from the stomach to the small intestine, β-glucan can only be degraded by oxidation [53]. Thus, β-glucan can activate gut-associated lymphoid tissue to exert an immunomodulatory effect. During the inflammation process, gut-associated lymphoid tissue significantly affects the elimination of inflammatory responses in comparison to the immune systems. When β-glucan enters the cecum, colon, and rectum, β-glucan is used by intestinal flora for fermentation. Ingestion of β-glucan promotes the production of more short-chain fatty acids and a lowering of pH in the cecum and colon, whereby the large amount of short-chain fatty acids produced via fermentation are advantageous for preventing inflammatory intestinal diseases [54]. Moreover, β-glucan can produce higher concentrations of butyrate than other soluble dietary fibers [55]. Studies have found that many dietary fibers can activate pattern recognition receptors of the intestinal immune cells and regulate the intestinal immune response and intestinal barrier function [56,57]. Miriam et al. studied the β-glucan–intestinal epithelial cell–dendritic cell–T cell immunization pathway and proved that soluble dietary fibers such as β-glucan can affect the human body’s immune system by stimulating the human mucosa [56]. Wang et al. found that β-glucan with a higher molecular weight was able to alter the gut microbiota. Moreover, our previous research found that the number-average molecular weights of HBBG, barley β-glucan, and oat β-glucan are 461,600, 201,900, and 30,500 g/mol, respectively.

In conclusion, our results demonstrate that HBBG alleviated DSS-induced UC in mice. After the introduction of HBBG to the diet, the DAI score and colonic MPO concentrations were decreased. Colonic atrophy and pathological damage to colon tissue were prevented. These results indicate that HBBG can enhance the barrier function of colonic epithelium, promote the production of anti-inflammatory cytokines (IL-10), reduce the production of proinflammatory cytokines (IL-1β, IL-6, and TNF-α), and adjust the intestinal microbiome structure. These results suggest that HBBG has the potential to serve as an effective anti-UC therapy.

## 4. Materials and Methods

### 4.1. Materials and Reagents

DSS (molecular weight 36,000–50,000) was purchased from MP Biomedicals, LLC (Solon, OH, USA). HBBG extracted from highland barley, and fecal occult blood test kits were provided by Shanghai Yuduo Biotechnology Company (Shanghai, China). The extraction of β-glucan was slightly modified from Westernlund’s method [58]. Highland barley was ground into powder with a mill. Isopropanol and petroleum ether were used to degrease. Thermostable alpha-amylase and trypsin were used to hydrolyze starch and remove proteins. The β-glucan was precipitated at 4 °C by using 60% ethanol solution. The insoluble residue was then dissolved in water and precipitated in 20% (NH_4_)_2_SO_4_. The insoluble residue was dissolved in water again and then dialyzed in distilled water. β-Glucan content was determined using the β-Glucanase Assay Kit, purchased from Neogen Bio-Scientific Technology Co., Ltd. (Shanghai, China). HBBG powder contains 81.21% ± 1.05% β-glucan. MPO, IL-1β, IL-6, IL-10, and TNF-α assay kits were obtained from Wuhan Boster Biological Technology, Ltd. (Wuhan, China). The HiScript II Q RT SuperMix qPCR kit was purchased from Vazyme Biotech Co., Ltd. (Nanjing, China). HieffTM qPCR SYBR^®^ Green Master Mix (No ROX) was purchased from Shanghai Yisheng Biotechnology Co., Ltd. (Shanghai, China).

### 4.2. MICE and Experimental Design

**Figure 7:**
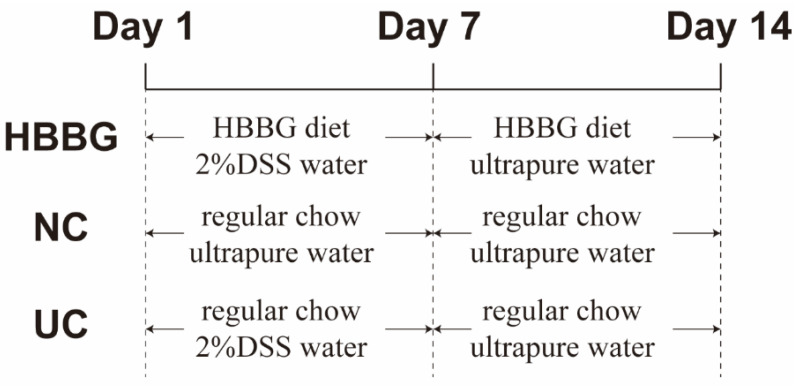
Diagrammatic representation of the 14 day experimental procedure.

In this study, 8 week old specific pathogen-free (SPF) male C57BL/6J mice were purchased from the Comparative Medicine Center of Yangzhou University (Yangzhou, China). The mice were kept in the Experimental Animal Center of Nanjing Agricultural University. All mice were allowed to drink ultrapure water and eat regular chow for 1 week to adapt to the environment. After adaptation, the 18 mice were allocated evenly to the NC, UC, and HBBG groups (*n* = 6 mice per group). The mice in the UC and HBBG groups were provided with 2% DSS dissolved in the water from days 1 to 7 to induce UC. The mice in the HBBG group were provided with an HBBG diet from days 1 to 14. And the mice in NC and UC groups were provided with regular chow. The composition of the experimental diet is shown in Table S2. The general health, body weight, food intake, and water intake of the mice were monitored from days 1 to 14. On the 14th day, the mice were anesthetized. The 14 day experimental procedure is shown in Figure 7. The mice were sacrificed by dislocating the neck. Serum samples were obtained by centrifuging the blood at 3000× *g* for 20 min. Cecal contents of all mice were placed in liquid nitrogen immediately and then stored in the −80 °C environment for extracting DNA and intestinal microbial analysis. After measuring the length, part of the colon tissue was quickly frozen in liquid nitrogen and stored in a −80 °C refrigerator for further experiments. Another part of the colon tissue was treated with 4% formaldehyde buffer for histological examination. All experiments were approved by the Laboratory Animal Welfare and Ethics Committee of Nanjing Agricultural University.

### 4.3. Assessment of DAI

The DAI scoring rules were slightly modified from Murthy et al. [59]. The DAI score is influenced by weight change, stool consistency, and results of the fecal occult blood test (FOBT). The specific scoring rules are shown in Table 1. The DAI value is the total of the three scores. An FOBT kit based on the *o*-toluidine method was used. Briefly, if the kit reagent did not turn a blue-green color after 3 min, it was considered a negative (−) result. If the reagent turned blue within 30–60 s, the sample was weakly positive (+). If the reagent immediately turned blue-green, it was considered positive (++). If the reagent immediately turned dark blue, it was strongly positive (+++).

### 4.4. Histopathological Analysis

An approximately 2 cm length of colon was removed 1 cm from the anus and treated with 4% paraformaldehyde solution for histopathological analysis. The histopathological scoring criteria were slightly modified from Murthy’s method [59]. The specific scoring rules are shown in Table 2.

### 4.5. Intestinal Permeability Test

According to Steedman’s method, Alcian blue staining can be used to study mucin changes of the colon tissue [60]. The intestinal permeability test was slightly modified from previously reported methods [61,62]. After fasting for 12 h, fluorescein isothiocyanate-dextran (FITC-Dextran) was administered intragastrically to the mice at a dose of 0.6 mg/g body weight. After 4 h, blood was collected and treated with anticoagulant, and the serum was collected by centrifugation at 3000× *g* for 12 min. The fluorescence intensity of the serum was measured at 490/520 nm (excitation/emission) with a spectrophotometer. The concentration of FITC-Dextran (and, thus, the intestinal permeability) was calculated using a standard curve.

### 4.6. RNA Isolation and RT-qPCR

Total RNA from the colon samples was extracted using the TRIzol method. The RNA was reverse-transcribed into single-stranded cDNA using the HiScript II Q RT SuperMix for qPCR kit (Vazyme Biotech Co., Ltd., Nanjing China). The RT-qPCR solutions were prepared according to the instructions of the HieffTM qPCR SYBR^®^ Green Master Mix kit (Yisheng Biotechnology Co., Ltd., Shanghai, China), and RT-qPCR was carried out using the Step OneTM Real-Time PCR system (ABI StepOne Plus, Applied Biosystems, Foster City, CA, USA). The transcriptional levels of genes encoding claudin-1, occludin, and ZO-1 were calculated using the 2^−ΔΔCT^ method, with GAPDH used as a reference housekeeping gene [11]. The primer sequences are listed in Table 3.

### 4.7. Determination of the Content of MPO in Colon Tissue

Colonic MPO concentrations were determined according to a modified version of the procedure reported by Chassaing et al. [63]. A 10% homogenate of each mouse colon tissue sample was prepared using a tissue homogenizer. The MPO concentration in the colon tissue of each group was measured using an MPO assay kit.

### 4.8. The Level of Cytokines in Serum

The concentrations of four cytokines in mouse serum samples were detected as per the experimental procedures provided by the ELISA kits.

### 4.9. Analysis of Intestinal Flora

Fecal microbial genome DNA was extracted using a QIAamp DNA Stool Mini Kit (Qiagen, Hilden, Germany). The variable region 4 (V4) of the 16S rRNA gene was amplified using the primer pair 520F/802R (5′-AYTGGGYDTAAAGNG-3′ and 5′-TACNVGGGTATCTAATCC-3′) [64]. The sequencing of the 16S rRNA V4 regions was performed by Shanghai Personal Biotechnology Co., Ltd. on the Illumina MiSeq platform.

### 4.10. Statistical Analysis

Data management and analysis were performed using SPSS 25, and results are expressed as the mean ± standard error of the mean (SEM). A one-way analysis of variance (ANOVA), followed by Tukey’s post hoc test, was conducted to determine whether any significant differences existed among different groups. A *p*-value <0.05 was considered statistically significant.

## Figures and Tables

**Table 1:** Criteria for scoring disease activity index.

Weight Loss	Stool Consistency	Occult Blood or Gross Bleeding	Score
0	Normal	negative (−)	0
1–5	Loose stools	positive (+)	1
5–10	Loose stools	positive (++)	2
10–15	Diarrhea	positive (+++)	3
>15	Diarrhea	Gross bleeding	4

Notes: normal stools = well-formed pellets; loose stools = pasty stool that does not stick to the anus; diarrhea = liquid stools that stick to the anus.

**Table 2:** Histological score.

Crypt Damage	Range of Lesion (%)	Degree of Inflammatory Infiltration	Depth of Lesions	Score
None	0%	None	None	0
Loss of bottom one-third of the crypt	1–25%	Mild	Mucosal layer	1
Loss of bottom two-thirds of the crypt	26–50%	Moderate	Submucosa	2
Loss of entire crypt with the surface epithelium remaining intact	51–75%	Severe	Muscular layer and serosal layer	3
Loss of the entire crypt and surface epithelium	76–100%	Severe	Muscular layer and serosal layer	4

**Table 3:** Primer Sequence used in RT-qPCR.

Gene	Upstream Primer (5′–3′)	Downstream Primer (5′–3′)
claudin-1	CGGGCAGATACAGTGCAAAG	ACTTCATGCCAATGGTGGAC
occludin	ATGTCCGGCCGATGCTCTC	TTTGGCTGCTCTTGGGTCTGTA
ZO-1	TTTTTGACAGGGGGAGTGG	TGCTGCAGAGGTCAAAGTTCAAG
MUC2	ATGCCCACCTCCTCAAAGAC	GTAGTTTCCGTTGGAACAGTGAA
GAPDH	TGCACCACCAACTGCTTAG	GGATGCAGGGATGATGTTC

## Data Availability

The data presented in this study are available on request from the corresponding author. The data are not publicly available due to continuity of the experiment.

## Supplementary Materials

Table S1: Comparison between highland barley and other cereals; Table S2: Composition of experimental diets.

## Sample Availability

Sample of highland barley β-glucan is available from the authors.
