# Supplementary material for: β-Glucan Extracted from Highland Barley Alleviates Dextran Sulfate Sodium-Induced Ulcerative Colitis in C57BL/6J Mice
Source: Molecules. 2021 Sep 25;26(19):5812. doi: 10.3390/molecules26195812 (PMC8510048; doi:10.3390/molecules26195812)
Supplement: Supplementary file 1 [file molecules-26-05812-s001.zip › molecules-1345998-supplementary.pdf]

Table S1 Comparison between highland barley and other cereals

ingredients	Summary of research results
Starch	Compared to wheat (65-75%), corn (64-78%), rice (~90%) and barley (51-77%), highland barley had less starch (49.14-68.62%).
Protein	Compared to rice, wheat, sorghum and corn, highland barley had more protein (6.35-23.4%).
Lipid	Highland barley had 1.18-3.09% crude lipid.
Fiber	Highland barley contained 15.01-21.45% fibers, higher than most cereals.
Mineral	Some minerals in highland barley such as Ca (350.7-975.5mg/kg), P (3032.75mg/kg), Fe (6.32-93.7mg/kg), Cu (3.64-6.02mg/kg), Zn (16.8-30.3mg/kg), Mn (14.49-20.29mg/kg) and Se (0.007-0.323mg/kg) were higher than in corn and the content of Fe was even higher than wheat and rice.
Vitamins	Highland barley had about 0.0389 mg/g-0.1084 mg/g Vitamin B, and 0.3-0.8 mg/100g Vitamin E which was higher than wheat.

Table S2 Composition of experimental diets.

Ingredient	Regular chow(g)	HBBG diet(g)
Casein	200	200
L-cystine	3	3
Corn starch	315	215
Maltodextrin	35	35
Sucrose	350	350
Cellulose	50	50
Soybean oil	25	25
Lard	20	20
Mineral mixture	10	10
Dicalcium phosphate	13	13
Calcium carbonate	5.5	5.5
Sodium citrate	16.5	16.5
Vitamin mixture	10	10
Choline bitartrate	2	2
Pigment	0.05	0.05
β-glucan	0	100
